# Genome-Wide Identification of the *WOX* Gene Family in Three *Populus* Species and Expression Profiling of *Populus euphratica* and *Populus pruinosa* Under Abiotic Stresses

**DOI:** 10.3390/ijms27135999

**Published:** 2026-07-03

**Authors:** Chen Qiu, Xinyue Long, Zhongshuai Gai, Xiaoli Han, Jia Song, Yuqi Yang, Jianhao Sun, Zhijun Li

**Affiliations:** 1 State Key Laboratory Incubation Base for Conservation and Utilization of Bio-Resource in Tarim Basin, Alar 843300, China; 2 College of Life Science and Technology, Tarim University, Alar 843300, China; 3 Desert Poplar Research Center, Tarim University, Alar 843300, China

**Keywords:** *Populus euphratica*, *Populus pruinosa*, *Populus deltoides*, *WOX* gene family, drought stress, salt stress

## Abstract

The WUSCHEL-related homeobox (*WOX*) gene family plays crucial roles in plant growth, development, and stress responses. In this study, a comprehensive genome-wide analysis of *WOX* genes was conducted in three *Populus* species, *P. euphratica*, *P. pruinosa*, and *P. deltoides*. A total of 16, 16, and 21 *WOX* genes were identified, respectively, and classified into three clades (ancient, intermediate, and WUS/modern) based on phylogenetic relationships. Structural analyses revealed highly conserved homeodomain motifs and similar exon–intron organizations, indicating strong evolutionary conservation. Furthermore, synteny analyses demonstrated that whole-genome duplication or segmental duplication events were the primary drivers of *WOX* gene family expansion, with most duplicated gene pairs undergoing purifying selection. Promoter analysis identified abundant cis-acting elements related to light responsiveness, hormone signaling, and stress responses. Notably, transcriptomic profiling during seed germination under drought and salt stress revealed distinct interspecific expression patterns and temporal dynamics between the two desert poplars. Specifically, members such as *PeWox11* and *PpWox2* were significantly induced, suggesting their potential involvement in abiotic stress adaptation. These findings provide comprehensive insights into the evolutionary conservation and divergence of *WOX* genes in *Populus*, establishing a theoretical foundation for the molecular breeding of stress-tolerant woody plants.

## 1. Introduction

The WUSCHEL-related homeobox (*WOX*) gene family is a plant-specific group of transcription factors belonging to the homeobox superfamily [1]. WOX proteins are defined by a highly conserved homeodomain—typically consisting of 60–66 amino acids—that folds into a characteristic helix–loop–helix–turn–helix structure [2]. This structural domain facilitates the specific recognition and binding of target DNA sequences, thereby orchestrating the transcription of downstream genes [3,4]. Based on phylogenetic relationships, the *WOX* gene family is generally classified into three major clades: the WUS/modern, intermediate, and ancient clade [5,6]. Among these, lower plants (such as green algae) contain only the ancient clade, while higher plants (such as seed plants) include all three clades [7,8,9].

As important regulators of the plant growth and development, WOX proteins orchestrate a wide array of physiological processes throughout plant ontogeny [10,11]. These processes encompass embryo initiation, the maintenance of meristematic stem cell niches, organogenesis, and the fine-tuned regulation of cell division and differentiation [12,13,14]. In the model plant *Arabidopsis thaliana*, *WOX1* and *WOX3* coordinate cell proliferation within their expression domains and surrounding tissues to promote leaf growth [15]. Furthermore, *WOX2* facilitates early embryogenesis by modulating *PIN1*-mediated auxin transport, while *WOX8* and *WOX9* are essential for ensuring precise embryonic patterning and maturation [16,17]. Beyond early development, *WOX14* plays a specialized role during inflorescence axis maturation by promoting the differentiation and lignification of vascular cells, thereby contributing significantly to the structural integrity and overall morphogenesis of the plant [18]. To date, *WOX* genes also have increasingly been recognized as critical mediators of plant responses to abiotic stressors. Previous studies have demonstrated that specific *WOX* members enhance plant adaptability to adverse environmental conditions, such as drought and salinity, by modulating root architecture and the expression of stress-responsive genes. For instance, the overexpression of *PagWOX11/12a* from hybrid poplar 84K (*Populus alba* × *Populus glandulosa*) has been shown to augment root biomass and significantly enhance drought tolerance in transgenic poplar plants [19]. Additionally, *PagWOX11/12a* positively regulates salt tolerance in poplar by transcriptionally activating the expression of the *PagCYP736A12* gene [20]. In *Malus domestica*, overexpression of *MdWOX13-1* has been associated with increased callus weight and enhanced ROS scavenging under drought conditions [21]. Similar protective roles have been observed in cereal crops; for example, *OsWOX11* enhances drought resistance in rice by regulating root hair development [22], while *OsWOX13* mediates drought responses by activating the promoters of the stress-responsive genes *OsDREB1A* and *OsDREB1F* [23]. Moreover, several *WOX* genes in rice exhibit responsive expression patterns to salt and cold stress, suggesting their involvement in multifaceted abiotic stress tolerance [24]. Collectively, these findings indicate that *WOX* genes play important roles in coordinating plant development and environmental adaptation.

The genus *Populus* served as a premier model system for investigating growth, development, and environmental adaptation in woody plants [25]. Within this genus, *P. euphratica* and *P. pruinosa* were typical desert riparian tree species that had long adapted to extreme environments characterized by drought and salinity, and therefore exhibited strong stress tolerance [26,27,28,29]. By contrast, *P. deltoides*, which serves as the primary source of raw material for the poplar industry, exhibits relatively limited tolerance to such environmental stressors [30]. Despite the known importance of WOX genes, their evolutionary divergence between stress-tolerant desert poplars and stress-sensitive poplars remains largely unexplored. Furthermore, while seed germination is the most vulnerable and critical stage for the establishment of desert riparian forests, the specific responses of WOX genes to abiotic stress during this phase are still poorly understood. To bridge these knowledge gaps, WOX gene family members were systematically identified and compared utilizing the genomic data of *P. euphratica*, *P. pruinosa*, and *P. deltoides*. Furthermore, their expression patterns and potential functions were characterized by integrating transcriptomic data from *P. euphratica* and *P. pruinosa* during seed germination under drought and salt stress conditions. These results will contribute to a deeper understanding of the roles of the *WOX* gene family in developmental regulation and environmental adaptation in *Populus* and provide a theoretical basis for molecular breeding aimed at improving stress tolerance.

## 2. Results

### 2.1. Identification and Characterization of WOX Family Members

**Figure 1:**
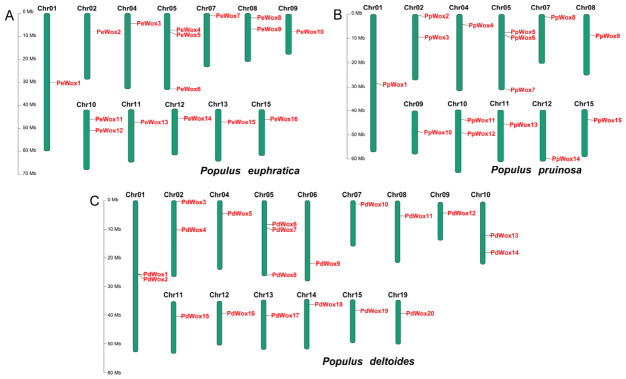
Genomic distribution of *WOX* genes across chromosomes of (**A**) *P. euphratica*, (**B**) *P. pruinosa*, and (**C**) *P. deltoides*.

In this research, 16, 16, and 21 *WOX* family members were identified in the genomes of *P. euphratica*, *P. pruinosa*, and *P. deltoides*, respectively. Based on their chromosomal distribution, these genes were designated as PeWox1-PeWox16, PpWox1-PpWox16, and PdWox1-PdWox21. It was observed that one to two WOX members were distributed across the majority of chromosomes, whereas no WOX genes were detected on 7, 8, and 4 chromosomes in *P. euphratica*, *P. pruinosa*, and *P. deltoides*, respectively (Figure 1). The analysis of physicochemical properties (Table S1) revealed that the encoded proteins of this gene family across the three species are composed of 152 to 481 amino acids. Their relative molecular weights were calculated to range from 17,706.75 to 53,549.24 Da, and their theoretical isoelectric points (pI) were determined to be between 5.33 and 9.3. The instability indices were evaluated at 40.72 to 73.4, classifying them as unstable proteins (index > 40). Furthermore, the grand average of hydropathicity (GRAVY) values were calculated to be entirely below zero (ranging from −0.385 to −1.119), which indicates that these proteins are hydrophilic in nature. Regarding secondary structure predictions (Table S2), all identified proteins consist of three characteristic structural elements: alpha helices, extended strands, and random coils. Among them, the random coil was identified as the predominant component, accounting for 61.9% to 82% of the structure, followed by alpha helix, whereas extended strand represented the lowest proportion. Finally, subcellular localization predictions demonstrated that 96% of the WOX members are localized within the nucleus, with the exceptions being PdWox2 and PdWox3 (Table S1).

### 2.2. Phylogenetic Analysis of Populus WOX Proteins

**Figure 2:**
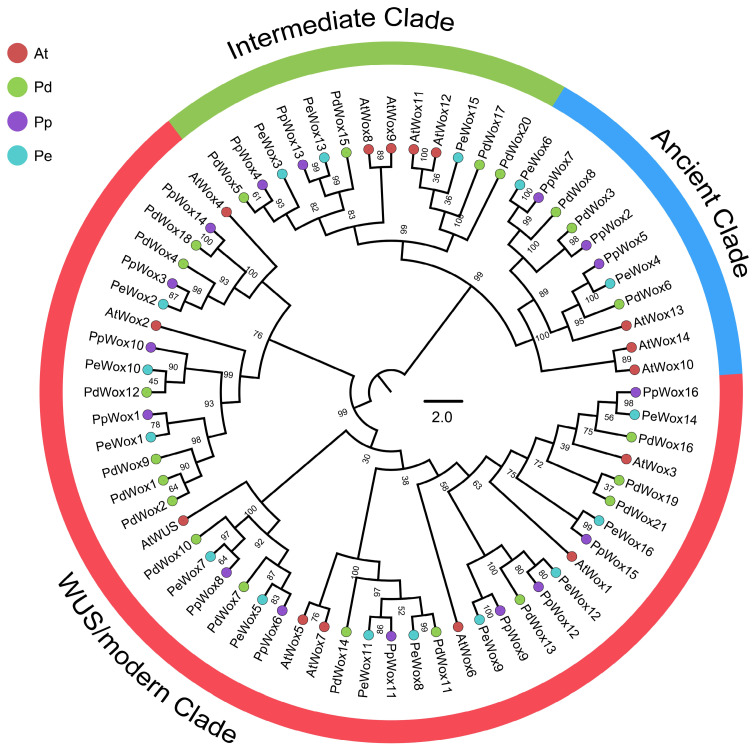
Phylogenetic tree of WOX gene family among *A. thaliana*, *P. euphratica*, *P. pruinosa*, and *P. deltoides*. Branch support was assessed using bootstrap values based on 1000 replicates.

To clarify the homologous evolutionary relationships among *WOX* gene family members in *P. euphratica*, *P. pruinosa*, *P. deltoides*, and *Arabidopsis thaliana*, a phylogenetic tree was constructed based on the amino acid sequences of WOX proteins (Figure 2 and Figure S1). With reference to the established classification of WOX family proteins in *A. thaliana*, the WOX family genes from the three *Populus* species were divided into three subfamilies: the ancient, intermediate, and WUS/modern clade. Among them, the WUS/modern clade was found to be the largest, comprising 44 genes, including 14 *PdWox* genes, 11 *PpWox* genes, 11 *PeWox* genes, and 8 *AtWox* genes. Furthermore, the intermediate clade contained 13 genes, including 4 *PdWox* genes, 2 *PpWox* genes, 3 *PeWox* genes, and 4 *AtWox* genes, whereas the ancient clade comprised 11 genes, including 3 *PdWox* genes, 3 *PpWox* genes, 2 *PeWox* genes, and 3 *AtWox* genes.

### 2.3. Analysis of Conserved Motif and Gene Structure

**Figure 3:**
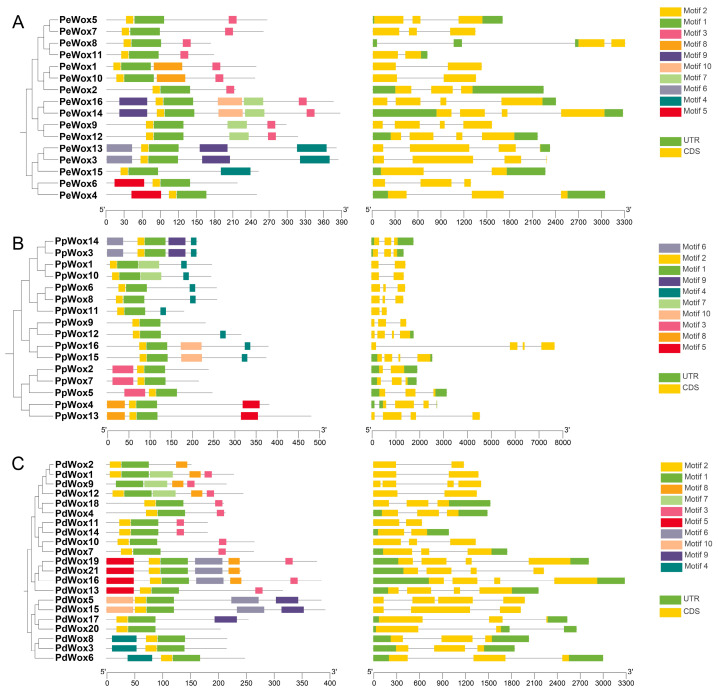
Phylogenetic relationships, motif distributions, and gene structure of (**A**) *P. euphratica*, (**B**) *P. pruinosa*, and (**C**) *P. deltoides WOX* Genes.

To investigate the structural characteristics of the *WOX* gene family in *P. euphratica*, *P. pruinosa,* and *P. deltoides*, the conserved motif composition of the encoded proteins and the gene structures were systematically compared and analyzed. Based on MEME analysis, 10 conserved motifs, designated Motif 1 to Motif 10, were predicted among the 53 *WOX* genes identified in *Populus* (Figure 3). The lengths of these motifs were determined to vary from 13 to 50 amino acids. Across all examined species, Motif 1 and Motif 2 (representing the HD domain), were found to be present and closely linked in all *WOX* genes, with the sole exception of *PdWox9*, indicating that these motifs were highly conserved within the *WOX* gene family. Furthermore, Motif 3 in *P. euphratica*, Motif 4 in *P. pruinosa*, and Motif 3 in *P. deltoides* were identified as the distinctive WUS-box motifs (TLXLFPXX), which are considered hallmark features of the WUS/modern clade members; however, this characteristic motif was observed to be absent in PdWox2. Gene structure analysis showed that the numbers of exons and introns among the 53 *WOX* genes were relatively similar, ranging from 2 to 4 and from 1 to 4, respectively. Notably, untranslated regions (UTRs) were found to be completely absent in twenty-one genes (comprising 5 in *P. euphratica*, 8 in *P. pruinosa*, and 8 in *P. deltoides*). It was also detected that only 5′ UTRs were possessed by *PeWox3*, *PeWox8*, and *PdWox21*, whereas solely 3′ UTRs were retained in *PeWox11*, *PeWox13*, *PeWox16*, and *PpWox12*. Conversely, both 5′ and 3′ UTRs were observed to be simultaneously present in all the remaining genes.

### 2.4. Synteny Analysis of Populus WOX Gene Family

**Figure 4:**
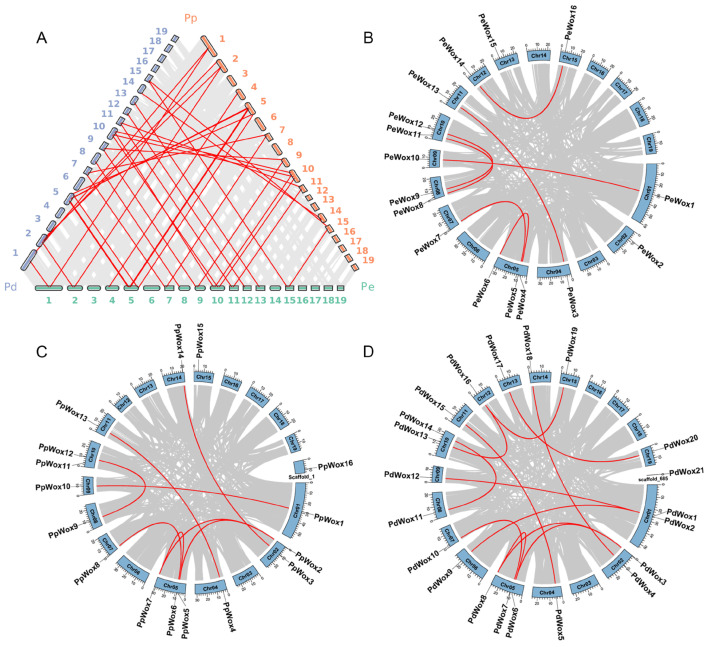
Analysis of collinearity among *Populus WOX* gene family. (**A**) *P. euphratica*, *P. pruinosa*, and *P. deltoides*; (**B**) intraspecific collinearity in *P. euphratica*; (**C**) intraspecific collinearity in *P. pruinosa*; (**D**) intraspecific collinearity in *P. deltoides*. The red lines highlight collinear WOX pairs.

To investigate the evolutionary relationships and expansion patterns of the *WOX* gene family across the three *Populus* species, both interspecific and intraspecific synteny analyses were performed in *P. euphratica*, *P. pruinosa*, and *P. deltoides*. In the results, a total of 13 syntenic gene pairs were identified between *P. euphratica* and *P. pruinosa*, and 14 syntenic gene pairs were identified between *P. euphratica* and *P. deltoides*, as well as between *P. pruinosa* and *P. deltoides* (Figure 4A). Additionally, regarding intraspecific synteny, 7, 9, and 12 collinear gene pairs were observed within the genome of *P. euphratica*, *P. pruinosa* and *P. deltoides*, respectively (Figure 4B–D). Furthermore, selection pressure analysis showed that the Ka/Ks ratios of both intraspecific paralogous pairs and interspecific orthologous pairs were less than 1 (ranging from 0.00 to 0.93), indicating that the WOX family has undergone intense purifying selection throughout its evolutionary history, emphasizing its high functional conservation (Table S3). In addition, analysis of duplication types within the WOX family indicated that the expansion of this gene family across the three *Populus* species was primarily associated with WGD or segmental duplication events (Table 1 and Table S4). For instance, 19 out of 21 duplicated gene pairs in *P. euphratica*, 14 out of 16 in *P. pruinosa*, and all 16 pairs in *P. deltoides* were attributed to this mechanism. Conversely, proximal and dispersed duplication events accounted for only a minor fraction of the total.

### 2.5. Cis-Acting Element Analysis

**Figure 5:**
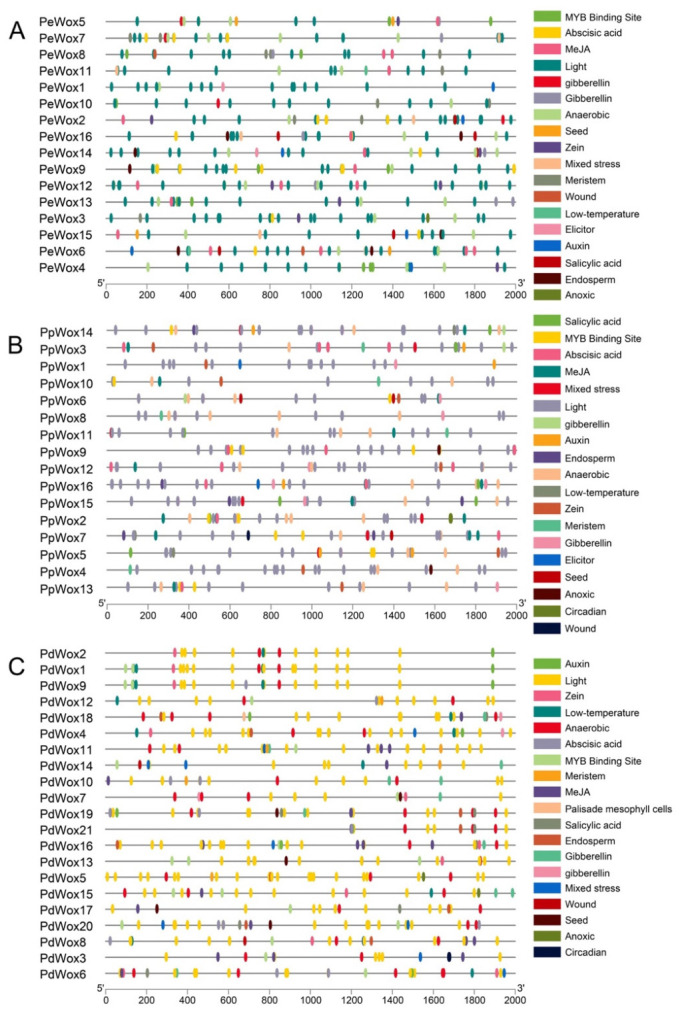
Predicted cis-acting elements in the 2000 bp upstream regions of (**A**) *P. euphratica*, (**B**) *P. pruinosa*, and (**C**) *P. deltoides WOX* Genes.

To explore the potential regulatory mechanisms of the *WOX* gene family in *Populus*, putative cis-acting regulatory elements in the promoter regions of *WOX* genes from *P. euphratica*, *P. pruinosa*, and *P. deltoides* were predicted using the 2 kb genomic sequences upstream of the translation start codon. The results showed that the promoter regions of *WOX* genes were enriched in diverse types of cis-acting elements. Among them, light-responsive elements, anaerobic induction-related elements, abscisic acid (ABA)-responsive elements, and methyl jasmonate (MeJA)-responsive elements were widely distributed in all three species (Figure 5). Further analysis revealed substantial variation in the number of cis-acting elements among different *WOX* genes. In *P. deltoides*, *PdWox7* contained the fewest cis-acting elements, with only 12 identified, followed by *PdWox21* and *PpWox14*, each harboring 14 elements. In contrast, the promoter region of *PeWox9* contained the largest number of cis-acting elements, with a total of 37 detected, followed by *PdWox4* and *PeWox6*, which contained 34 and 33 elements, respectively. These findings suggested that the promoter regions of *Populus WOX* genes possess abundant and diverse regulatory elements, implying that this gene family may participate in plant growth, development, and environmental stress responses through complex regulatory networks.

### 2.6. Expression Analysis of WOX Genes in P. euphratica and P. pruinosa Seeds Under Severe Drought and Salt Stress

**Figure 6:**
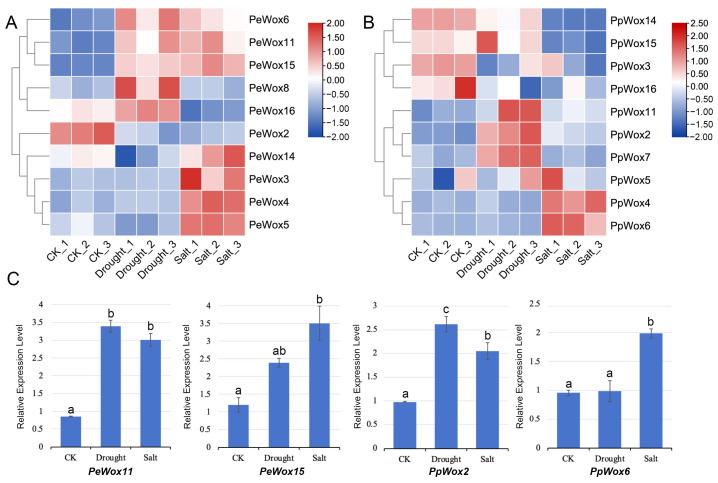
Expression heatmap of the *PeWox* (**A**) and *PpWox* (**B**) gene family during seed germination under severe drought and salt stress; (**C**) The relative expression level of *PeWox11*, *PeWox15*, *PpWox2*, and *PpWox6* in drought and salt stress. Significant differences were determined by ANOVA with the 0.05 *p*-value threshold. Different lowercase letters indicate significant differences between treatments (*p* < 0.05).

As typical desert riparian forest species, *P. euphratica* and *P. pruinosa* have developed long-term adaptations to extreme environmental conditions, including aridity and high salinity. Consequently, to further elucidate the potential functions of the *WOX* gene family in stress adaptation, the expression profiles were systematically analyzed utilizing transcriptomic datasets derived from the seed germination stage under drought and salt stress treatments. It was demonstrated by the results that distinct interspecific expression patterns were exhibited by the *WOX* gene family between the two *Populus* species. In *P. euphratica*, *PeWox11*, and *PeWox15* were observed to be significantly upregulated under both drought and salt stress conditions (FoldChange > 1.5, *p* < 0.05, Table S5). Furthermore, it was detected that *PeWox6*, *PeWox8*, and *PeWox12* were induced exclusively by drought stress, while *PeWox3* and *PeWox5* were found to be upregulated solely under salt stress (Figure 6A). Conversely, in *P. pruinosa*, *PpWox2*, and *PpWox11* were significantly upregulated under both drought and salt stress conditions (FoldChange > 1.5, *p* < 0.05, Table S5), with a more pronounced expression increase under drought stress than under salt stress. In addition, the upregulation of *PpWox4*, *PpWox6*, and *PpWox13* was identified under salt stress (Figure 6B). Furthermore, to validate the accuracy of the RNA-Seq data, four genes (*PeWox11*, *PeWox15*, *PpWox2*, and *PpWox6*) were selected for qRT-PCR analysis in drought and salt stress (Table S6). The qRT-PCR results revealed expression trends consistent with FPKM values (Figure 6C), supporting the reliability of the transcriptome data. It is suggested by these findings that functional divergence may have occurred within the *WOX* gene family between these two desert *Populus* species.

## 3. Discussion

The *WOX* transcription factors serve as crucial regulators in plant growth, development, and stress responses [31]. In this study, 16, 16, and 21 *WOX* members were identified in *P. euphratica*, *P. pruinosa*, and *P. deltoides*, respectively. The total number of WOX members varies considerably across plant species, with some retaining only about ten members [13,32,33] while others have expanded to over thirty [10,34]. Such numerical disparity likely arises from species-specific genome duplication events, chromosomal rearrangements, and divergent evolutionary selection pressures. Predictive analysis of subcellular localization indicated that 96% of the identified WOX members were localized within the nucleus, which was highly consistent with their canonical function as transcription factors that regulate gene expression [1]. Notably, PdWox2 and PdWox3 were predicted to exhibit non-nuclear localization, suggesting they may fulfill unique functional roles within the cytoplasm or be governed by specialized regulatory mechanisms. Furthermore, phylogenetic analysis categorized the 53 *Populus WOX* genes into three classical clades: the ancient, intermediate, and WUS/modern clades. The WUS/modern clade emerged as the largest group, suggesting that it has undergone substantial expansion within the *Populus* lineage. Structural characterization revealed the ubiquitous presence of Motif 1 and Motif 2 across almost all members; these two motifs correspond to the highly conserved homeodomain [32]. Additionally, the minimal variation in intron numbers among these genes further corroborates the evolutionary conservation of *Populus* WOX proteins.

Gene duplication is a fundamental evolutionary mechanism driving gene family expansion and the acquisition of novel functions [35,36]. The synteny analysis revealed that WGD or segmental duplication events served as the primary driving forces behind the expansion of the *WOX* gene family in these three *Populus* species. This finding is highly congruent with the extensive genomic rearrangements and polyploidization events previously documented in the *Populus* lineage [37]. While tandem and dispersed duplication events were also identified, their contributions to the overall expansion were relatively minor, thereby underscoring the critical role of large-scale genomic events. Furthermore, the evaluation of Ka/Ks ratios for both interspecific and intraspecific collinear gene pairs demonstrated that these values were consistently <1. This provides compelling evidence that the *WOX* gene family has predominantly been subjected to strong purifying selection, suggesting that the core biological functions of these genes have been highly conserved throughout evolution. Such stringent selective pressure is a hallmark of genes governing essential developmental processes.

Gene expression patterns are intricately governed by cis-acting regulatory elements within their promoter regions, which synergistically orchestrate responses to developmental signals and environmental stimuli. The promoter analysis revealed an abundance of cis-elements, including those responsive to light, anaerobic conditions, and phytohormones such as ABA and MeJA. These phytohormones play crucial roles in regulating plant stress responses to drought and salinity [38]. Previous studies have demonstrated that *WOX* genes are involved in diverse stress responses, such as modulating cellular ROS scavenging capacity [21] or enhancing drought sensitivity [39]. The quantitative and qualitative variations in cis-elements observed across the promoters of different *WOX* genes further imply a complex and finely tuned regulatory landscape, thereby enabling diversified and environment-specific transcriptional responses.

*P. euphratica* and *P. pruinosa* are iconic desert riparian tree species renowned for their exceptional tolerance to severe drought and high salinity, making them excellent models for studying stress adaptation. Seed germination, a highly vulnerable stage in the plant life cycle, demands robust molecular mechanisms for successful establishment under harsh conditions. Transcriptomic analysis showed that several WOX genes from both *P. euphratica* and *P. pruinosa* respond to drought and salt stress during seed germination. Among them, *PeWox11*, *PeWox15*, and *PpWox2* showed responsiveness to both drought and salt stress, suggesting that these genes may contribute to a core osmotic stress response. This is consistent with our promoter analysis, which revealed that these genes share multiple stress-responsive motifs, such as MeJA and Anaerobic-responsive elements. In contrast, the stress-specific induction of other WOX members implies further functional diversification within this gene family. For example, *PpWox6* was specifically induced by salt stress, which correlates with its unique enrichment of Abscisic acid-responsive and seed-specific elements. These patterns are consistent with findings in other species like saffron, where *WOX* genes show tissue-specific and stress-specific expression [40]. Collectively, these patterns indicate that the diversity and density of cis-elements in the promoter regions likely drive the functional specialization of WOX genes, reflecting the distinct strategies of these two desert *Populus* species for coping with arid and saline environments.

In conclusion, the comprehensive bioinformatic analysis of the *WOX* gene family in three *Populus* species, particularly focusing on *P. euphratica* and *P. pruinosa*, provides deep insights into their evolutionary conservation, structural features, and dynamic expression patterns under drought and salt stress during seed germination. The observed functional divergence and species-specific responses of *WOX* genes likely contribute to the remarkable adaptability of these desert *Populus* species to extreme environmental conditions. While this study provides a robust foundation for understanding *WOX* gene function, future research should integrate experimental validation, such as gene editing or overexpression studies, to confirm the specific roles of key *WOX* members. Furthermore, investigating their downstream targets and interacting proteins will elucidate the molecular mechanisms by which *WOX* genes orchestrate development and stress tolerance in these ecologically important trees.

## 4. Materials and Methods

### 4.1. Identification and Physicochemical Characterization of WOX Gene Family Members

To systematically identify the *WOX* transcription factor family, the whole-genome sequences of *P. euphratica* [41], *P. pruinosa* [27], and *P. deltoides* [42] were utilized. Initially, the Hidden Markov Model (HMM) profile corresponding to the WOX domain (PF00046) was retrieved from the Pfam database [43] (https://www.ebi.ac.uk/interpro/entry/pfam/#table, accessed on 13 January 2026). This profile was employed to scan the proteomes of the three *Populus* species using HMMER [44] (version 3.3.2; http://hmmer.org/, accessed on 13 January 2026) with an *E*-value threshold of <0.001. To refine the search, the identified candidate sequences were used to construct a species-specific HMM for a second round of rigorous screening (*E* < 0.001). Concurrently, a BLASTp (version 2.5.0+) search was performed using *A. thaliana* WOX protein [16] sequences as queries against the three *Populus* protein databases (E < 1 × 10^−5^). To ensure the reliability of the results, all candidate sequences were further validated for the presence of the conserved homeodomain via the SMART [45] (http://smart.embl-heidelberg.de/, accessed on 18 January 2026), InterPro [46] (https://www.ebi.ac.uk/interpro/, accessed on 18 January 2026), and NCBI-CDD databases [47] (https://www.ncbi.nlm.nih.gov/cdd/, accessed on 18 January 2026); sequences lacking the characteristic domain were manually removed. The physicochemical attributes of the identified WOX proteins, including amino acid length, molecular weight, pI, instability index, and GRAVY, were characterized using the Expasy-ProtParam tool [48] (https://web.expasy.org/protparam/, accessed on 20 January 2026). Furthermore, the subcellular localization of the WOX proteins was predicted using the WoLF PSORT online platform [49] (https://wolfpsort.hgc.jp/, accessed on 20 January 2026).

### 4.2. Phylogenetic Reconstruction and Classification

To evaluate the evolutionary relationships and classify the identified genes, a phylogenetic tree was constructed using the protein sequences of *P. euphratica*, *P. pruinosa*, *P. deltoides*, and *A. thaliana*. Multiple sequence alignment was performed using the MUSCLE algorithm with default parameters. Subsequently, the phylogenetic relationships were analyzed using MEGA12 (version 12.0.11) [50] via both Neighbor-Joining and Maximum Likelihood methods. The reliability of the tree branches was assessed with 1000 bootstrap replicates. The final phylogenetic tree was visualized and annotated using the iTOL v7 online platform [51] (https://itol.embl.de/, accessed on 5 February 2026) to distinguish the different evolutionary clades.

### 4.3. Gene Structure and Conserved Motif Identification

The conserved motifs within the WOX protein sequences were identified using the MEME suite [52] (version 5.5.8; https://meme-suite.org/meme/tools/meme, accessed on 28 January 2026), with the maximum number of motifs set to 10. Concurrently, the exon–intron structures of the *WOX* genes were extracted from the GFF/GTF annotation files of the three *Populus* species using TBtools-II (version 2.476) [53]. The integration of the phylogenetic tree, conserved motifs, and gene structures was visualized using the “Gene Structure View” module in TBtools-II to analyze the structural conservation and divergence among the members.

### 4.4. Synteny, Gene Duplication, and Evolutionary Selection Analysis

For the synteny analysis, collinear blocks both within each species and across the three species were identified using the JCVI toolkit [54] (https://github.com/tanghaibao/jcvi, accessed on 30 January 2026). Interspecific collinearity relationships were visualized through the jcvi.graphics.karyotype module, while intraspecific gene duplication events were mapped and displayed using TBtools-II. To further elucidate the expansion mechanisms of the *WOX* gene family, the MCScanX was utilized to identify and classify the specific gene duplication types across the complete genomes of the three *Populus* species. In addition, Ka, Ks, and Ka/Ks ratios for both paralogous and orthologous *WOX* gene pairs were calculated using the “Simple Ka/Ks Calculator” program integrated within TBtools-II.

### 4.5. Cis-Acting Element Prediction

To explore the potential transcriptional regulation of *WOX* genes in response to environmental stimuli, the 2 kb promoter regions upstream of the translation initiation codon were retrieved using TBtools-II. These sequences were submitted to the PlantCARE database [55] (http://bioinformatics.psb.ugent.be/webtools/plantcare/html/, accessed on 25 January 2026) to identify putative cis-acting regulatory elements. The distribution and types of these elements were subsequently classified and visualized using TBtools-II.

### 4.6. RNA-Seq for Drought and Salt Stress

To investigate the transcriptional response of *WOX* genes to abiotic stresses, RNA-seq datasets from our previous studies [56,57] were used to analyze *P. euphratica* and *P. pruinosa* during seed germination under drought and salt stress. Seeds were germinated in Petri dishes with filter paper under a 16 h light (30 °C)/8 h dark (25 °C) photoperiod. Based on a 7-day preliminary test where germination dropped below 50%, 15% PEG 6000 and 0.3 mol/L NaCl were selected to simulate severe drought and salt stress. For the formal experiment, whole seedlings (>20 per sample) were harvested 4 days after treatment—when cotyledons were fully expanded—and immediately stored at −80 °C for sequencing.

To ensure the high resolution and accuracy of the expression profiles, all transcriptomic data were re-analyzed using the high-quality reference genomes and annotations of *P. euphratica* [41] and *P. pruinosa* [27]. Initially, the raw sequencing reads were processed using trimmomatic (version 0.39) [58] to remove adapters and low-quality sequences, thereby yielding clean reads. Subsequently, these clean reads were aligned to their respective reference genomes using HISAT2 (version 2.2.1) [59] with default parameters. The quantification of genes was performed using StringTie (version 2.1.7) [60] with fragments per kilobase of transcript per million fragments mapped (FPKM). WOX genes with FPKM values lower than 1 across all samples were excluded, and the remaining genes were used for heatmap visualization generated with TBtools-II. The differential expression analysis was performed using the (DESeq2) package (version 1.50.2) ased on the raw count data. The normalization strategy followed the default. Genes with a statistical threshold of *p* < 0.05 and an absolute value of ∣log2FoldChange∣ > 0.585 (equivalent to a FoldChange > 1.5) were identified as significantly differentially expressed genes.

### 4.7. qRT-PCR Validation

The qRT-PCR experiments were conducted on the mRNA from *P. euphratica* and *P. pruinosa* samples collected during seed germination under drought and salt stress. Total RNA was reverse-transcribed using the SPARKscript RT Plus Kit with gDNA Eraser (SparkJade, Jinan, China). Reactions were run in triplicate on an ABI 7500 real-time PCR system (Thermo Fisher Scientific, Waltham, MA, USA) using SYBR Green qPCR Mix (SparkJade, Jinan, China). The *PeActin* sequence was used as the endogenous control, and the primers used are listed in Table S6. The CT values were analyzed using the 2^−∆∆CT^ method.

## Figures and Tables

**Table 1:** *WOX* gene family duplication types in three *Populus* species.

Species	Proximal	Dispersed	WGD or Segmental
*P. euphratica*	1	1	19
*P. pruinosa*	0	2	14
*P. deltoides*	0	0	16

## Data Availability

Sequence data from this article can be found in the National Genomics Data Center (https://ngdc.cncb.ac.cn/, accessed on 5 January 2026) with the project number CRA005566 and the NCBI database (https://www.ncbi.nlm.nih.gov/, accessed on 5 January 2026) with the project number PRJNA484685.

## Supplementary Materials

The following supporting information can be downloaded at: https://www.mdpi.com/article/10.3390/ijms27135999/s1.

## Abbreviations

The following abbreviations are used in this manuscript:

WOX	WUS homeobox-containing
pI	theoretical isoelectric points
GRAVY	grand average of hydropathicity
UTRs	untranslated regions
ABA	abscisic acid
MeJA	methyl jasmonate
HMM	Hidden Markov Model
FPKM	fragments per kilobase of transcript per million fragments mapped

## References

[1] Graaff E.v.d., Laux T., Rensing S.A. (2009). The WUS homeobox-containing (WOX) protein family. Genome Biol..

[2] Gehring W.J., Qian Y.Q., Billeter M., Furukubo-Tokunaga K., Schier A.F., Resendez-Perez D., Affolter M., Otting G., Wüthrich K. (1994). Homeodomain-DNA recognition. Cell.

[3] Gehring W.J., Müller M., Affolter M., Percival-Smith A., Billeter M., Qian Y.Q., Otting G., Wüthrich K. (1990). The structure of the homeodomain and its functional implications. Trends Genet..

[4] Mukherjee K., Brocchieri L., Bürglin T.R. (2009). A comprehensive classification and evolutionary analysis of plant homeobox genes. Mol. Biol. Evol..

[5] Lin H., Niu L., McHale N.A., Ohme-Takagi M., Mysore K.S., Tadege M. (2013). Evolutionarily conserved repressive activity of WOX proteins mediates leaf blade outgrowth and floral organ development in plants. Proc. Natl. Acad. Sci. USA.

[6] Dolzblasz A., Nardmann J., Clerici E., Causier B., Graaff E.v.d., Chen J., Davies B., Werr W., Laux T. (2016). Stem Cell Regulation by Arabidopsis WOX Genes. Mol. Plant.

[7] Alvarez J.M., Bueno N., Cañas R.A., Avila C., Cánovas F.M., Ordás R.J. (2018). Analysis of the *WUSCHEL-RELATED HOMEOBOX* gene family in *Pinus pinaster*: New insights into the gene family evolution. Plant Physiol. Biochem..

[8] Nardmann J., Werr W. (2012). The invention of WUS-like stem cell-promoting functions in plants predates leptosporangiate ferns. Plant Mol. Biol..

[9] Segatto A.L.A., Thompson C.E., Freitas L.B. (2016). Molecular evolution analysis of *WUSCHEL-related homeobox* transcription factor family reveals functional divergence among clades in the homeobox region. Dev. Genes Evol..

[10] Hao Q., Zhang L., Yang Y., Shan Z., Zhou X.-A. (2019). Genome–wide analysis of the WOX gene family and function exploration of GmWOX18 in soybean. Plants.

[11] Rasheed H., Shi L., Winarsih C., Jakada B.H., Chai R., Huang H. (2024). Plant Growth Regulators: An Overview of WOX Gene Family. Plants.

[12] Jha P., Ochatt S.J., Kumar V. (2020). *WUSCHEL*: A master regulator in plant growth signaling. Plant Cell Rep..

[13] Xu J., Hu Z., Chen S., Tang J., Chen L., Chen P., Cai N., Xu Y. (2025). Transcriptome-wide identification and characterization of *WUSCHEL-related homeobox* (WOX) gene family in *Pinus yunnanensis*. BMC Genom..

[14] Wu C.-C., Li F.-W., Kramer E.M. (2019). Large-scale phylogenomic analysis suggests three ancient superclades of the WUSCHEL-RELATED HOMEOBOX transcription factor family in plants. PLoS ONE.

[15] Nakata M., Matsumoto N., Tsugeki R., Rikirsch E., Laux T., Okada K. (2012). Roles of the middle domain-specific *WUSCHEL-RELATED HOMEOBOX* genes in early development of leaves in *Arabidopsis*. Plant Cell.

[16] Haecker A., Gross-Hardt R., Geiges B., Sarkar A., Breuninger H., Herrmann M., Laux T. (2004). Expression dynamics of WOX genes mark cell fate decisions during early embryonic patterning in *Arabidopsis thaliana*. Development.

[17] Ueda M., Zhang Z., Laux T. (2011). Transcriptional activation of *Arabidopsis* axis patterning genes WOX8/9 links zygote polarity to embryo development. Dev. Cell.

[18] Denis E., Kbiri N., Mary V., Claisse G., Silva N.C.E., Kreis M., Deveaux Y. (2017). WOX14 promotes bioactive gibberellin synthesis and vascular cell differentiation in *Arabidopsis*. Plant J..

[19] Wang L.-Q., Li Z., Wen S.-S., Wang J.-N., Zhao S.-T., Lu M.-Z. (2020). WUSCHEL-related homeobox gene *PagWOX11/12a* responds to drought stress by enhancing root elongation and biomass growth in poplar. J. Exp. Bot..

[20] Wang L.-Q., Wen S.-S., Wang R., Wang C., Gao B., Lu M.-Z. (2021). PagWOX11/12a activates PagCYP736A12 gene that facilitates salt tolerance in poplar. Plant Biotechnol. J..

[21] Lv J., Feng Y., Jiang L., Zhang G., Wu T., Zhang X., Xu X., Wang Y., Han Z. (2023). Genome-wide identification of WOX family members in nine Rosaceae species and a functional analysis of *MdWOX13-1* in drought resistance. Plant Sci..

[22] Cheng S., Zhou D.-X., Zhao Y. (2016). *WUSCHEL*-related homeobox gene *WOX11* increases rice drought resistance by controlling root hair formation and root system development. Plant Signal. Behav..

[23] Minh-Thu P.-T., Kim J.S., Chae S., Jun K.M., Lee G.-S., Kim D.-E., Cheong J.-J., Song S.I., Nahm B.H., Kim Y.-K. (2018). A WUSCHEL Homeobox Transcription Factor, OsWOX13, Enhances Drought Tolerance and Triggers Early Flowering in Rice. Mol. Cells.

[24] Cheng S., Huang Y., Zhu N., Zhao Y. (2014). The rice *WUSCHEL*-related homeobox genes are involved in reproductive organ development, hormone signaling and abiotic stress response. Gene.

[25] Shi T., Zhang X., Hou Y., Jia C., Dan X., Zhang Y., Jiang Y., Lai Q., Feng J., Feng J. (2024). The super-pangenome of *Populus* unveils genomic facets for its adaptation and diversification in widespread forest trees. Mol. Plant.

[26] Jiao P., Wu Z., Wang X., Jiang Z., Wang Y., Liu H., Qin R., Li Z. (2021). Short-term transcriptomic responses of *Populus euphratica* roots and leaves to drought stress. J. For. Res..

[27] Sun J., Xu J., Qiu C., Zhai J., Zhang S., Zhang X., Wu Z., Li Z. (2024). The chromosome-scale genome and population genomics reveal the adaptative evolution of *Populus pruinosa* to desertification environment. Hortic. Res..

[28] Huang Z., Zhai J., Li Z., Yu L. (2024). *Populus euphratica* has stronger regrowth ability than *Populus pruinosa* under salinity stress. Physiol. Plant.

[29] Li G., Chen Q., Bai Q., Feng Y., Mao K., Yang M., He L., Liu M., Liu J., Wan D. (2023). LncRNA expression analysis by comparative transcriptomics among closely related poplars and their regulatory roles in response to salt stress. Tree Physiol..

[30] Zhao T. (1992). *Populus deltoides* and Its Hybrid in Poplar Cultivation in China and in the World. World For. Res..

[31] Li M., Wang R., Liu Z., Wu X., Wang J. (2019). Genome-wide identification and analysis of the *WUSCHEL*-related homeobox (WOX) gene family in allotetraploid *Brassica napus* reveals changes in WOX genes during polyploidization. BMC Genom..

[32] Lyu Q., Chen S., Wang X., Yuan Y., Zhang H., Liang W., Cheng H., Deng Z. (2026). Genome-Wide Identification and Expression Analysis of the WOX Family Reveals Potential Roles in Stem Development of *Euphorbia hirta*. Plants.

[33] Zhang X., Zong J., Liu J., Yin J., Zhang D. (2010). Genome-wide analysis of WOX gene family in rice, sorghum, maize, Arabidopsis and poplar. J. Integr. Plant Biol..

[34] Rathour M., Sharma A., Kaur A., Upadhyay S.K. (2020). Genome-wide characterization and expression and co-expression analysis suggested diverse functions of WOX genes in bread wheat. Heliyon.

[35] Lynch M., Conery J.S. (2000). The evolutionary fate and consequences of duplicate genes. Science.

[36] Panchy N., Lehti-Shiu M., Shiu S.-H. (2016). Evolution of Gene Duplication in Plants. Plant Physiol..

[37] Ma T., Wang J., Zhou G., Yue Z., Hu Q., Chen Y., Liu B., Qiu Q., Wang Z., Zhang J. (2013). Genomic insights into salt adaptation in a desert poplar. Nat. Commun..

[38] Zhu J.-K. (2016). Abiotic Stress Signaling and Responses in Plants. Cell.

[39] Tang Y., Li H., Guan Y., Li S., Xun C., Dong Y., Huo R., Guo Y., Bao X., Pei E. (2020). Genome-Wide Identification of the Physic Nut WUSCHEL-Related Homeobox Gene Family and Functional Analysis of the Abiotic Stress Responsive Gene *JcWOX5*. Front. Genet..

[40] Tao Y., Li J., Chen J., Xi X., Yang S., Qiu F., Zhang X., Feng M., Qian X., Li L. (2026). Genome-wide identification and characterization of WOX gene family in saffron (*Crocus sativus* L.) and their roles in stress response, development and callus formation. Front. Plant Sci..

[41] Zhang S., Wu Z., Ma D., Zhai J., Han X., Jiang Z., Liu S., Xu J., Jiao P., Li Z. (2022). Chromosome-scale assemblies of the male and female *Populus euphratica* genomes reveal the molecular basis of sex determination and sexual dimorphism. Commun. Biol..

[42] Xue L., Wu H., Chen Y., Li X., Hou J., Lu J., Wei S., Dai X., Olson M.S., Liu J. (2020). Evidences for a role of two Y-specific genes in sex determination in *Populus deltoides*. Nat. Commun..

[43] Mistry J., Chuguransky S., Williams L., Qureshi M., Salazar G.A., Sonnhammer E.L.L., Tosatto S.C.E., Paladin L., Raj S., Richardson L.J. (2021). Pfam: The protein families database in 2021. Nucleic Acids Res..

[44] Eddy S.R. (2011). Accelerated Profile HMM Searches. PLoS Comput. Biol..

[45] Letunic I., Bork P. (2026). SMART v10: Three decades of the protein domain annotation resource. Nucleic Acids Res..

[46] Blum M., Andreeva A., Florentino L.C., Chuguransky S.R., Grego T., Hobbs E., Pinto B.L., Orr A., Paysan-Lafosse T., Ponamareva I. (2025). InterPro: The protein sequence classification resource in 2025. Nucleic Acids Res..

[47] Wang J., Chitsaz F., Derbyshire M.K., Gonzales N.R., Gwadz M., Lu S., Marchler G.H., Song J.S., Thanki N., Yamashita R.A. (2023). The conserved domain database in 2023. Nucleic Acids Res..

[48] Gasteiger E., Hoogland C., Gattiker A., Duvaud S.e., Wilkins M.R., Appel R.D., Bairoch A. (2005). Protein Identification and Analysis Tools on the ExPASy Server.

[49] Horton P., Park K.-J., Obayashi T., Fujita N., Harada H., Adams-Collier C.J., Nakai K. (2007). WoLF PSORT: Protein localization predictor. Nucleic Acids Res..

[50] Kumar S., Stecher G., Suleski M., Sanderford M., Sharma S., Tamura K. (2024). MEGA12: Molecular Evolutionary Genetic Analysis Version 12 for Adaptive and Green Computing. Mol. Biol. Evol..

[51] Letunic I., Bork P. (2024). Interactive Tree of Life (iTOL) v6: Recent updates to the phylogenetic tree display and annotation tool. Nucleic Acids Res..

[52] Bailey T.L., Johnson J., Grant C.E., Noble W.S. (2015). The MEME Suite. Nucleic Acids Res..

[53] Chen C., Chen H., Zhang Y., Thomas H.R., Frank M.H., He Y., Xia R. (2020). TBtools: An Integrative Toolkit Developed for Interactive Analyses of Big Biological Data. Mol. Plant.

[54] Tang H., Krishnakumar V., Zeng X., Xu Z., Taranto A., Lomas J.S., Zhang Y., Huang Y., Wang Y., Yim W.C. (2024). JCVI: A versatile toolkit for comparative genomics analysis. Imeta.

[55] Lescot M., Déhais P., Thijs G., Marchal K., Moreau Y., Peer Y.V.d., Rouzé P., Rombauts S. (2002). PlantCARE, a database of plant cis-acting regulatory elements and a portal to tools for in silico analysis of promoter sequences. Nucleic Acids Res..

[56] Sun J. (2024). Genomic-Based Investigation of *Populus pruinosa*’s Adaptive Evolution to Desert Environments. Ph.D. Thesis.

[57] Sun J., Xu J., Qu W., Han X., Qiu C., Gai Z., Zhai J., Qin R., Liu H., Wu Z. (2023). Genome-wide analysis of R2R3-MYB transcription factors reveals their differential responses to drought stress and ABA treatment in desert poplar (*Populus euphratica*). Gene.

[58] Bolger A.M., Lohse M., Usadel B. (2014). Trimmomatic: A flexible trimmer for Illumina sequence data. Bioinformatics.

[59] Pertea M., Kim D., Pertea G.M., Leek J.T., Salzberg S.L. (2016). Transcript-level expression analysis of RNA-seq experiments with HISAT, StringTie and Ballgown. Nat. Protoc..

[60] Kovaka S., Zimin A.V., Pertea G.M., Razaghi R., Salzberg S.L., Pertea M. (2019). Transcriptome assembly from long-read RNA-seq alignments with StringTie2. Genome Biol..

